# Fat-containing cells are eliminated during *Dictyostelium* development

**DOI:** 10.1242/bio.025478

**Published:** 2017-07-27

**Authors:** Jessica M. Kornke, Markus Maniak

**Affiliations:** Abteilung Zellbiologie, Universität Kassel, D-34109 Kassel, Germany

**Keywords:** Fatty acid metabolism, Fat storage, Lipid droplets, Development, Seipin, *Dictyostelium discoideum*

## Abstract

Triacylglycerol is a universal storage molecule for metabolic energy in living organisms. However, *Dictyostelium* amoebae, that have accumulated storage fat from added fatty acids do not progress through the starvation period preceding the development of the durable spore. Mutants deficient in genes of fat metabolism, such as *fcsA*, encoding a fatty acid activating enzyme, or *dgat1* and *dgat2*, specifying proteins that synthesize triacylglycerol, strongly increase their chances to contribute to the spore fraction of the developing fruiting body, but lose the ability to produce storage fat efficiently. *Dictyostelium* seipin, an orthologue of a human protein that in patients causes the complete loss of adipose tissue when mutated, does not quantitatively affect fat storage in the amoeba. *Dictyostelium seiP* knockout mutants have lipid droplets that are enlarged in size but reduced in number. These mutants are as vulnerable as the wild type when exposed to fatty acids during their vegetative growth phase, and do not efficiently enter the spore head in *Dictyostelium* development.

## Abstract (precis)

**Summary:** In contrast to many living organisms, storage fat is not beneficial for *Dictyostelium* cells when progressing through starvation and subsequent development of a dormant stage.

## INTRODUCTION

Fat storage has many well-known roles in biology. For warm-blooded animals living in the sea, like whales, or in cold climates, such as bears, it forms an insulating layer beneath the skin to protect the body from loss of thermal energy. At the same time, the fat tissue stores chemical energy that may be consumed, e.g. during hibernation. Because of its physico-chemical properties the main constituent of fat, triacylglycerol (TAG), is a weight- and volume-optimized form of energy depot due to the absence of bound water. This is, for instance, an obvious advantage for migratory birds. Even plants exploit these properties to provide their embryos with energy for germination, in excess to that what could be carried in a given volume by a sugar-based storage-molecule like starch.

In order to generate energy from TAG, the molecule needs to be broken down to its constituents, namely one glycerol molecule and three fatty acids. The latter are degraded by sequentially clipping off two-carbon atom units in a process called beta-oxidation. It predominantly takes place in mitochondria of animals, whereas plant seedlings use a specialized peroxisome-like organelle instead. In addition, the fat molecules do not only funnel into catabolism, but also serve as building blocks for cellular membranes, because only a few biochemical reactions need to be performed to convert TAG into a phospholipid. Taken together, these examples suggest that TAG is in general a useful molecule that is able to provide a selective advantage to the organism bearing it, under natural (i.e. non-civilized) conditions.

Fat storage has been addressed in a number of model organisms such as *Drosophila*, *Caenorhabditis*, and yeast, but little is known about this branch of metabolism in *Dictyostelium*. In the laboratory, *Dictyostelium* cells are mostly cultivated in so-called axenic liquid medium and, as a result, the cells are virtually free of storage fat. In contrast, *Dictyostelium* cells cultivated on bacteria as a food source synthesize storage fat (Long and Coe, 1974), which is packaged into lipid droplets (Matsuoka et al., 2003). Addition of a fluorescent fatty acid analogue to the medium also induces the formation of lipid droplets, albeit under more reproducible conditions (von Löhneysen et al., 2003). Because *Dictyostelium* cells tolerated palmitic acid especially well (Weeks, 1976), we established by mass spectrometry that this fatty acid was easily incorporated into TAG, and used the resulting lipid droplets to analyse the lipid and protein constituents, as well as the dynamics of their formation and degradation in vegetatively growing cells (Du et al., 2013).

In cells that have received palmitic acid, the growth rate was reported to be unaffected (Weeks, 1976). Because in the vegetative-phase cells drew no discernible advantage from their fat reserves, we resorted to analysing the developmental phase of *Dictyostelium*. Here, nutrient-depleted individual amoebae form an aggregate and later differentiate to form a fruiting body, where dead stalk cells support a bolus of spores which will be able to germinate and form the next generation. Unexpectedly, we found that cells bearing lipid storage droplets were out-competed by lean cells, so that they did not contribute much to spore formation and thus were missing in the next generation of amoebae. Moreover, our results show that this effect is lipid droplet-specific and cannot be explained by lipotoxicity of the added fatty acids.

## RESULTS

### Metabolic status controls the development of *Dictyostelium*

**Fig. 1.**
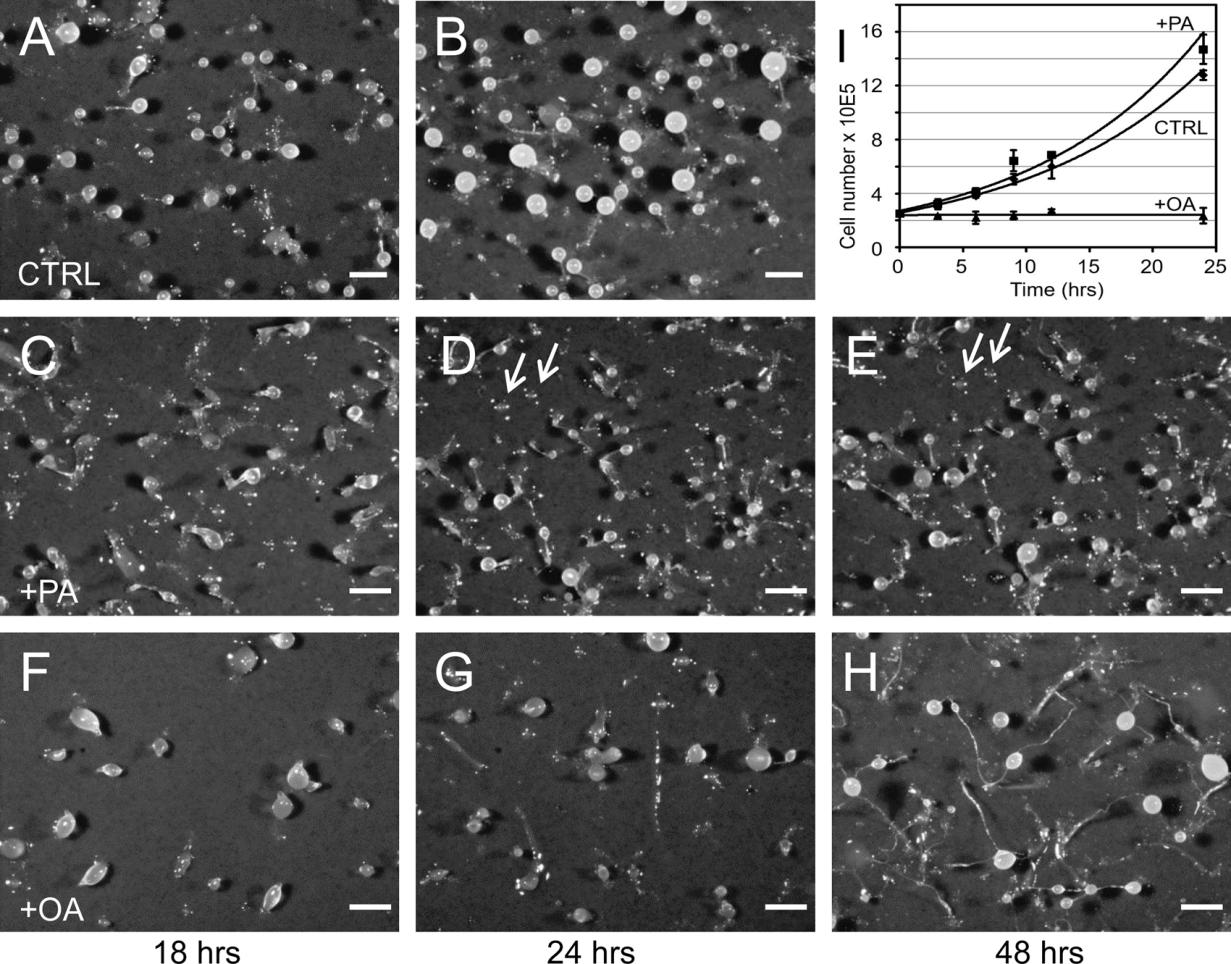
**Mild versus deleterious effects of fatty acids on *Dictyostelium* growth and development.** (A-H) *Dictyostelium* wild-type cells were allowed to develop on a moist black filter substrate and photographs were taken after the times indicated (h). Untreated cells (CTRL) are shown in panels A and B. When cells received palmitic acid (+PA) during the last 3 h before development was initiated (C-E), they formed smaller and fewer fruiting bodies (D), and aggregates were occasionally left behind on the surface that did not develop further after 24 h (arrows). Development was strongly delayed (F-H) by a pre-incubation with oleic acid (+OA) and mature fruiting bodies were found only after 48 h (H). Scale bars: 0.5 mm. (I) Samples were withdrawn from cells in growth phase at the intervals indicated in h and cell numbers were determined in a Coulter counter. Cultures that had received 200 µM of palmitic acid (+PA, squares) at t_0_ grew slightly faster than untreated controls (CTRL, diamonds), whereas cultures treated with 200 µM oleic acid (+OA, triangles) ceased growth immediately. The mean values (symbols) from 3 independent experiments are connected by a curve representing an exponential function. Error bars indicate mean±s.d.

Although palmitic acid treatment was previously not reported to seriously affect development (Weeks, 1976), we added 200 µM palmitic acid to the medium of growth phase cells, which results in the abundant formation of lipid droplets within 3 h (Du et al., 2013). The cells were washed in non-nutrient buffer and allowed to undergo their developmental program on a filter surface in a moist environment. After 18 h, these cells (Fig. 1C) slightly lagged behind the untreated control (Fig. 1A). At 24 h, mature fruiting bodies were formed, that were characterized by somewhat smaller heads and slightly thicker and shorter stalks (Fig. 1D) as compared to fruiting bodies derived from untreated cells (Fig. 1B). Although the number of fruiting bodies derived from palmitic acid-induced cells was 29% higher, the total number of spores recovered from their plates was only roughly 78% as compared to untreated controls, supporting the observation of smaller heads. Furthermore, it became clear, that many cells were left behind on the filter surface, which did not proceed further in development even in the subsequent 24 h period (Fig. 1E and D, arrows). In contrast, cells exposed to 200 µM oleate for only 3 h needed roughly 48 h to complete development and showed a branched network of excessively long thin stalks (Fig. 1F-H). Moreover, we found that oleic acid-treated vegetative cells ceased growth immediately (Fig. 1I), while a 3-h treatment with palmitic acid supports normal cell growth (Fig. 1I).

**Fig. 2.**
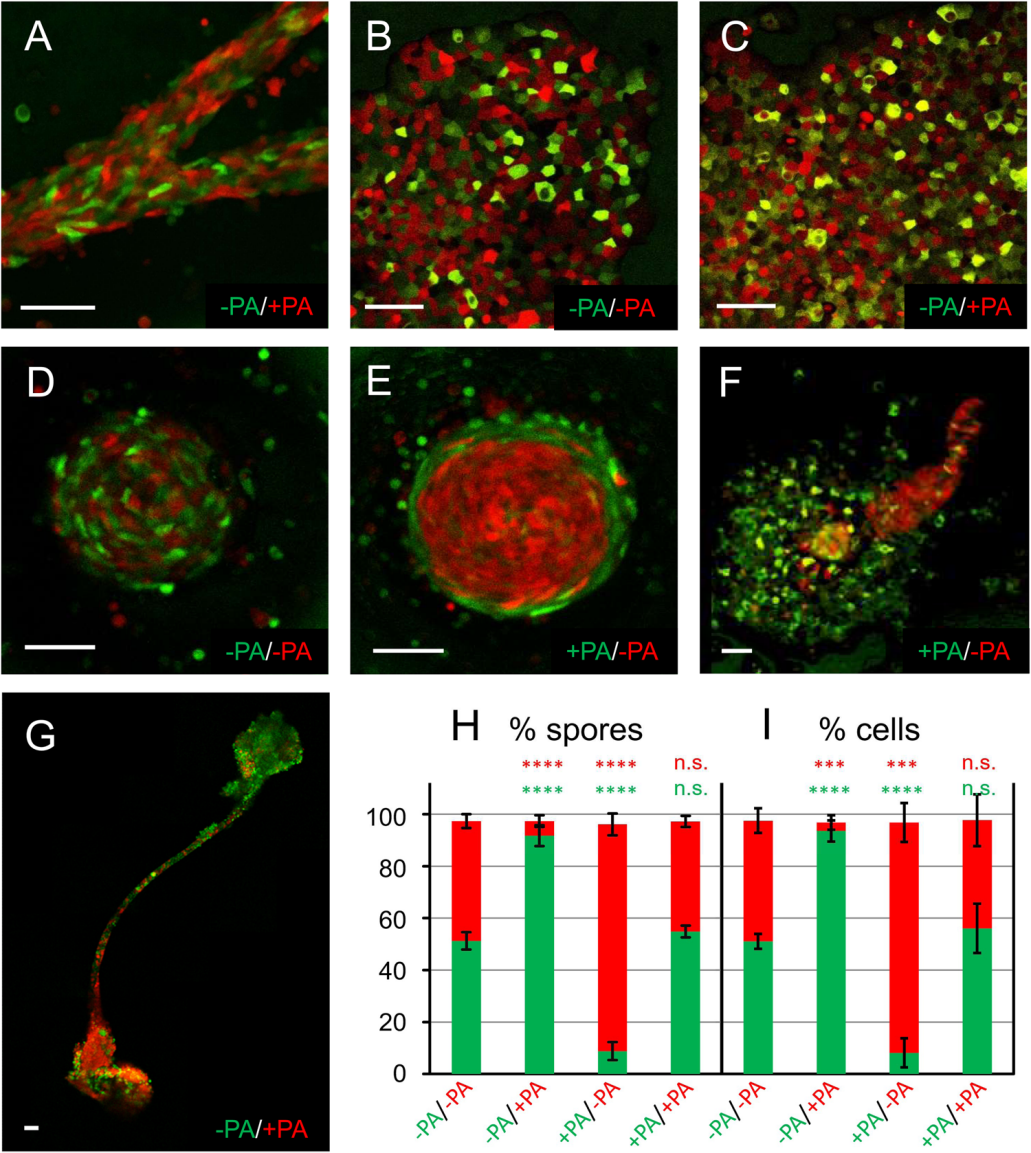
**Palmitic acid-treated cells do not contribute to the next generation.** (A-G) Cells expressing GFP or RFP, as indicated by the respective colour labelling, were incubated for 3 h in growth medium containing (+PA) or lacking (−PA) 200 µM palmitic acid and mixed with an equal number of cells of the other colour, washed immediately in non-nutrient buffer and allowed to develop on a moist agar surface. (A) In streams imaged by confocal microscopy the cells behaved largely similar. (B,C) In 12 h-old aggregates many PA-treated cells round up and are subsequently phagocytosed by untreated cells that keep their amoeboid shape (C), while this phenomenon is rare when all cells are untreated (B). In mounds, where cells are rotating around the centre, red and green cells are well intermingled when untreated (D), whereas cells that were preincubated with PA tend to sort out to the periphery (E). (F) A multicellular migratory structure consisting mostly of red untreated cells is seen to emanate from a field of PA-incubated green cells that remain at the original place of aggregation. For this image, eight confocal sections taken from different z-planes were superimposed. (G) Five overlapping confocal sections through a fruiting body stitched together to reveal the whole structure. Red fluorescent cells dominate in the basal plate (lower left) whereas green cells constitute the majority in the spore head (upper right). Scale bars: 50 µm (in A-G). (H) Bar diagram showing the percentage of red (RFP) and green (GFP) spores after mechanical dissociation of the spore head. The strain that had received the 3 h palmitic acid-treatment during the growth phase is indicated by +PA. For each bar, five mixing experiments were initiated and at least 100 spores were counted. Between 2 to 4% of spores were non-fluorescent and thus omitted from the graph. ****P<0.0001 for s.e.m. values as compared to the result when both strains were untreated (first bar); n.s., not significant. Panel I gives the results after counting cells that have germinated from the spore-heads roughly one week after harvesting. ***P<0.001. *N*=3 biological replicates. Otherwise abbreviations are as in (H).

Because palmitic acid-treated cells completed the development in the same time span as untreated cells (Fig. 1D), we decided to follow their fates in an experiment where the cells are mixed in a 50:50 ratio. Initially, the cells co-aggregated in streams with roughly equal efficiency (Fig. 2A) with only some fat cells remaining at the sides. Subsequently, however, three surprising observations were made. First, aggregates showed numerous round cells derived from the treated cell fraction, which we assume to be dead, because the corpses were internalized by neighbouring healthy cells from the untreated fraction (Fig. 2C). This process is also observed if both strains are untreated, albeit at a strongly reduced frequency (Fig. 2B). Secondly, in mounds that start rotating, no segregation occurs if both strains are untreated (Fig. 2D), whereas lean cells accumulate in the core of the mound and the lipid droplet-containing cells are sorted to the periphery (Fig. 2E). Apparently, many of the fat cells are left behind when a migratory stage emanates (Fig. 2F), which preferentially contains lean cells. Finally, the mature fruiting bodies mainly consist of untreated cells dominating in the spore head and the fat cells accumulated preferentially in the basal plate (Fig. 2G). To quantify this distribution spores were harvested and counted (Fig. 2H), demonstrating that untreated cells made up about 90% of the spore population. To further analyse whether the efficiency of propagation was altered by palmitic acid treatment, the spores were re-introduced into growth medium and cells were counted after germination. As seen in Fig. 2I, their quantitative distribution perfectly matched the numbers of spores counted before.

**Fig. 3.**
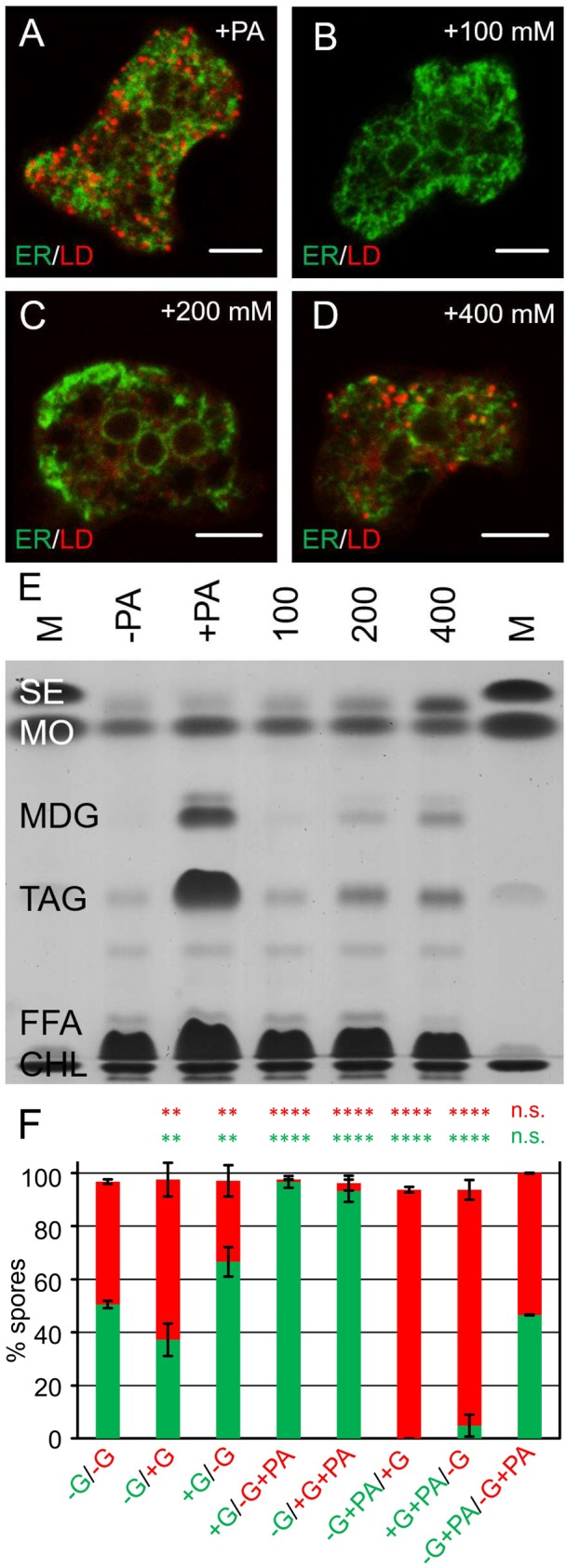
**Fatty acid-treatment overrules the effect of glucose on *Dictyostelium* development.** (A-D) Single confocal sections through fixed cells pre-incubated with 200 µM palmitic acid (A) or different concentrations of glucose as indicated (mM) (B-D). Lipid droplets (LD) are stained in red using the fat-specific dye LD540. The green counterstaining shows the ER as detected by a primary antibody directed against the protein disulfide isomerase (PDI) and a secondary antibody coupled to Oregon Green 488. Scale bars: 5 µm. (E) Thin layer chromatography resolving endogenous steryl-esters (SE), the ether-lipid monoalkyl-diacylglycerol (MDG), triacylglycerol (TAG), free fatty acids (FFA), and cholesterol (CHL) from lipid extracts of wild-type cells that have either received a 3-h treatment with 200 µM palmitic acid (+PA) or increasing amounts of glucose (from 100 to 400 mM) for the same time during growth phase. Cells from normal growth medium (containing 50 mM of glucose) are shown for comparison (−PA). Methyloleate (MO) was added as a tracer to account for possible loss of material during lipid preparation. M, lane with lipid markers. Two biological replicates were made. (F) Bar diagram representing spore numbers after development of RFP- or GFP-expressing wild-type cells originating from different cultivation conditions in the previous vegetative phase. Standard growth medium contained 50 mM glucose (+G) or was devoid (−G), together with (+PA) or in the absence (no label) of palmitic acid. *N*=3 biological replicates, otherwise the experiment was conducted as described in Fig. 2H.

Previously, glucose availability has been linked to developmental fate in *Dictyostelium* (Leach et al., 1973). In mixing experiments, cells grown in the absence of glucose sorted to the tip of the multicellular migratory stage, the slug, and ended up in the stalk; whereas cells that were provided with glucose predominated in the rear portion of the slug and subsequently became spores (Thompson and Kay, 2000). Therefore, we first investigated whether lipid droplets accumulated in glucose-fed cells (Fig. 3A-D) and found that at the highest concentration tested (Fig. 3D) some droplets appear. Thin layer chromatography revealed that these droplets did not contain much TAG, but rather were stores for steryl-esters and ether lipids (Fig. 3E).

First, we set out to recapitulate the effect of glucose in our cell mixing system and found that over 60% of cells that were grown in glucose containing medium became spores irrespective of the expressed fluorescent protein (bars 2 and 3 in Fig. 3F). This value does not reach the numbers published the original work from Leach et al. (1973), but the distribution is quite consistent with more recent experiments by Dubravcic et al. (2014). More relevant to our work, however, is the result of adding fatty acid to cells that were previously grown in a medium lacking glucose, which were then mixed with cells from normal medium. Under these conditions, the glucose-supported spore fate and PA-repression synergize, so that the resulting spores are essentially purely derived from the glucose-cultivated strain (bars 4 and 6 in Fig. 3F). However, in the converse experiment, where the strain grown in the presence of glucose was supplied with palmitic acid, it contributed only roughly 5% to the spore number (bars 5 and 7 in Fig. 3F) with the glucose-depleted strain making up the remaining 90%. This observation strongly indicates that the cell loss mediated by palmitic acid predominates any effect of glucose addition by far.

### Lipid droplet biogenesis constitutes a selective disadvantage

**Fig. 4.**
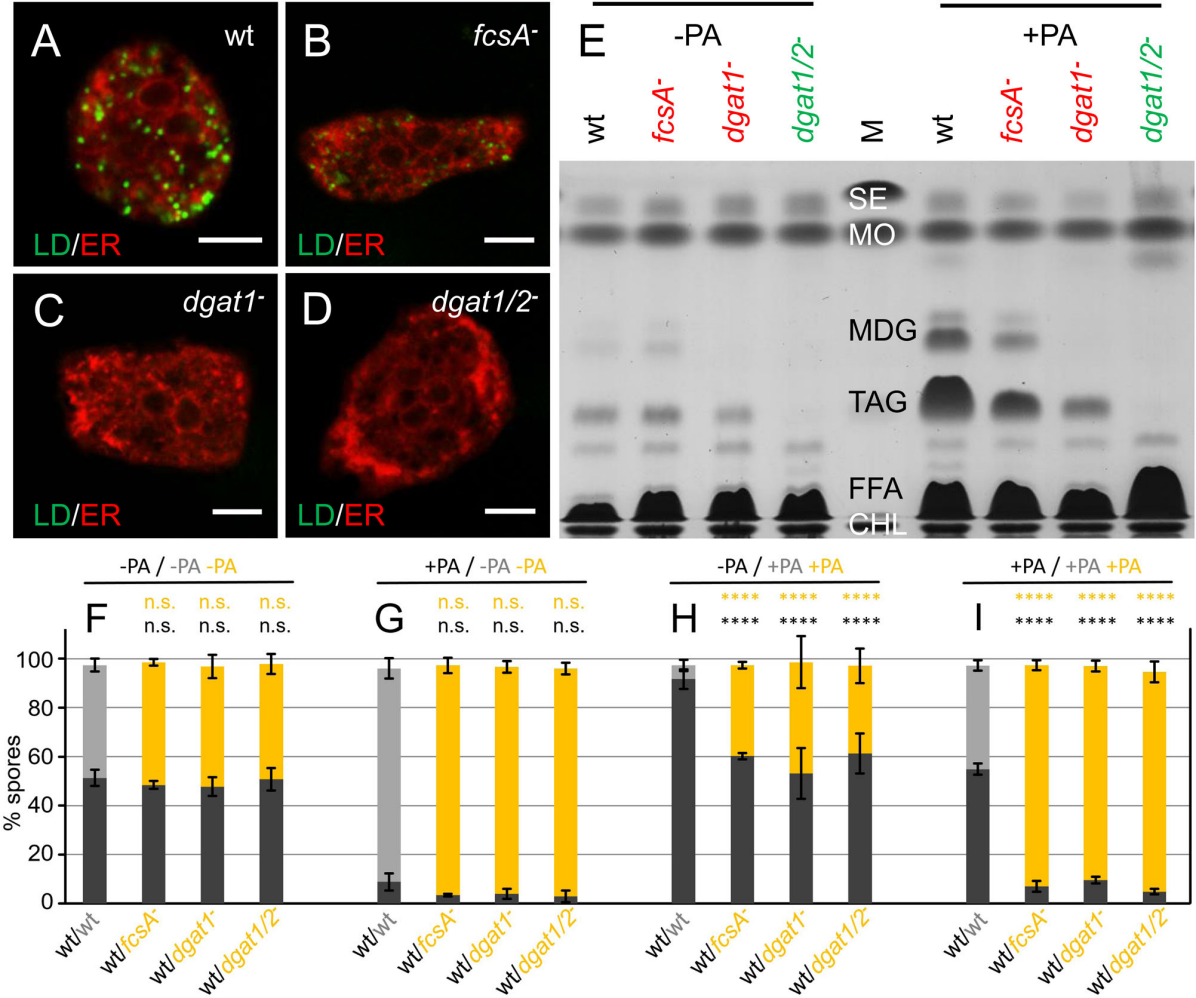
**Lean mutants survive development.** (A-D) Cells of the wild type (wt) and of different genotypes, as indicated, were incubated for 3 h in growth medium containing 200 µM palmitic acid to induce lipid droplet (LD) formation while addition of trace amounts of the fluorescent fatty acid analog C_1_-BODIPY-C_12_ served to stain them in green. The red counterstaining results from immunodetection of the ER-resident protein PDI as specified in Fig. 3, except that the secondary antibody was coupled with CY3 (red). Scale bar: 5 µm. (E) Three strains deficient in *fcsA*, *dgat1*, or *dgat1* and *dgat2* simultaneously, as well as the wild type (wt), expressing RFP or GFP as markers for fluorescence detection as indicated, were cultivated for 3 h in the absence (left) or presence (right) of palmitic acid (PA). Major lipid classes were analyzed by thin layer chromatography and compared to marker substances (M) as in Fig. 3E. Three biological replicates were made. (F-I) Bar diagram showing the percentage of wild type (wt, dark or light grey bars) and mutant (yellow bars) spores after harvesting. The genotype relating to lipid metabolism is indicated. For panel F, untreated cells were mixed in equal amounts and allowed to develop, whereas the wild type (dark grey, wt) was subjected to a 3-h palmitic acid incubation (+PA) in panel G. Conversely, the three mutants, and as a control the RFP-expressing wild type (light grey bar) in column 1, were exposed to the fatty acid (+PA) in (H), while in the last combination (I) all cells were grown in the presence of palmitic acid before undergoing development. ****P<0.0001 and always relates to the first bar in each panel, where both strains labelled by different fluorescent proteins carry an otherwise wild-typical genotype. Again, each mixing experiment was conducted 3 times and over 100 spores were counted.

Fatty acids added to the growth medium are internalized and concomitantly receive a coenzyme A moiety that renders them competent to enter lipid metabolism. In *Dictyostelium* this role is ascribed mainly to the FcsA protein (von Löhneysen et al., 2003). In times of fatty acid excess, three acyl chains are sequentially linked to one glycerol molecule, where the final step is performed by two acyltransferases, Dgat1 and Dgat2. A single mutant in the *dgat1* gene is clearly impaired in the production of triacylglycerol, and a double mutant lacking both enzymes is virtually unable to synthesize fat (Du et al., 2014). In microscopic analyses *fcsA^−^* cells (Fig. 4B) contained fewer lipid droplets than the wild type (Fig. 4A), which appeared to be absent in the *dgat^−^* mutants altogether (Fig. 4C,D). For subsequent quantitative analysis, the strains were transformed to express red fluorescent protein (RFP) or green fluorescent protein (GFP) labels and the lipid composition of each strain was documented both before and after palmitic acid addition by thin layer chromatography (Fig. 4E), revealing that there is a gradual decrease in the mutants' ability to generate TAG, starting with a strong reduction in *fcsA*^−^ cells, to the complete absence of TAG in the *dgat1/2^−^* double knockout.

During development, mutants and wild types each contributed roughly 50% of the spores if untreated (Fig. 4F), and as expected, the mutants also formed over 90% of the spores when mixed with palmitic acid-treated wild type (Fig. 4G). However, even when the mutant cells were subjected to the pre-incubation in palmitic acid-containing medium, they were able to form spores three to four times more efficiently than wild-type cells (Fig. 4H). Their strong dominance also continued when both stains received the fatty acid treatment (Fig. 4I) indicating that it is the biogenesis of lipid droplets and not the presence of palmitic acid alone, or another metabolite, that precludes the cells from becoming spores.

### A change in lipid droplet size and number does not affect cell fate

**Fig. 5.**
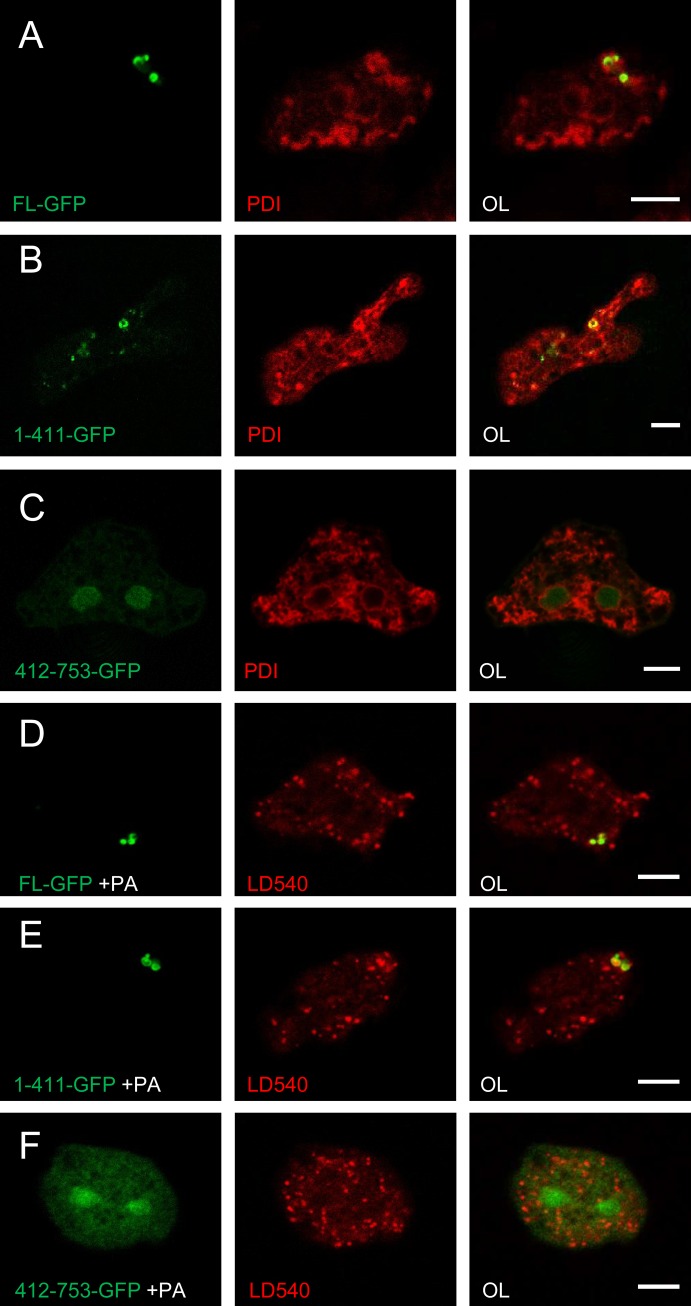
**Seipin associates with a subset of lipid droplets.** Confocal sections from fixed cells expressing either full-length seipin (FL in A,D), the seipin homologous region (amino acid 1-411, panels B,E), or the unrelated part (amino acid 412-753, in C,F) each tagged with GFP (green) at its respective C-terminus. Cells were left untreated (A-C) and the endoplasmic reticulum was revealed by immunofluorescence using an antibody directed against the protein-disulfide isomerase (PDI, red). Alternatively, the cells were incubated with palmitic acid (+PA, in D-F) to induce the formation of lipid droplets as stained by LD540 (red). Scale bars: 5 µm.

One of the proteins found in the *Dictyostelium* lipid droplet proteome is related to mammalian seipin (Du et al., 2013). Mutations in this protein cause the congenital form of Berardinelli-Seip lipodystrophy, characterized by the absence of fat tissue (Magré et al., 2001; Van Maldergem et al., 2002). Therefore, it appeared worthwhile to investigate the function of seipin in *Dictyostelium* cells. The *Dictyostelium seiP* gene (DDB0308581) encodes a protein of 753 amino acids, where the N-terminal half, up to position 411, is 31% identical to human seipin. If conserved amino acid exchanges are taken into account, the homology increases to 50%. The C-terminal domain reveals no relationship to other proteins and is characterized by a predominance of Asn residues (96 out of 342) that often occur as homo-polymers up to 25 residues long. Because the *Dictyostelium* seipin sequence features three predicted transmembrane domains, one of which is very close to the N-terminus of the protein, we fused a GFP reporter to the C-terminus of the full-length protein to observe its localization within the cell. The label was rare, but most prominent in regions of the cell that were rich in endoplasmic reticulum (Fig. 5A). This distribution was faithfully reproduced by the first 411 amino acids that were truly seipin-like (Fig. 5B). In contrast, the unrelated C-terminal sequence from position 412 to 753 was cytosolic, with some enrichment in the nucleus (Fig. 5C).

To analyse seipin's relation to lipid droplets (LDs), the GFP-expressing cells were pre-incubated with palmitic acid and the forming LDs were labelled by virtue of a fat-specific dye (Spandl et al., 2009). The C-terminal part of the molecule remained in cytoplasm and nucleus (Fig. 5F). Interestingly, only a small number of LDs were associated with the GFP-staining originating from full-length seipin (Fig. 5D) or its N-terminal half (Fig. 5E). Thus, like in yeast (Szymanski et al., 2007) it is restricted to foci and rings that could represent junctions between the ER and the growing LDs, rather than being distributed all over the ER membrane, as seen in a variety of mammalian cell types (Chen et al., 2009; Fei et al., 2011; Windpassinger et al., 2004).

**Fig. 6.**
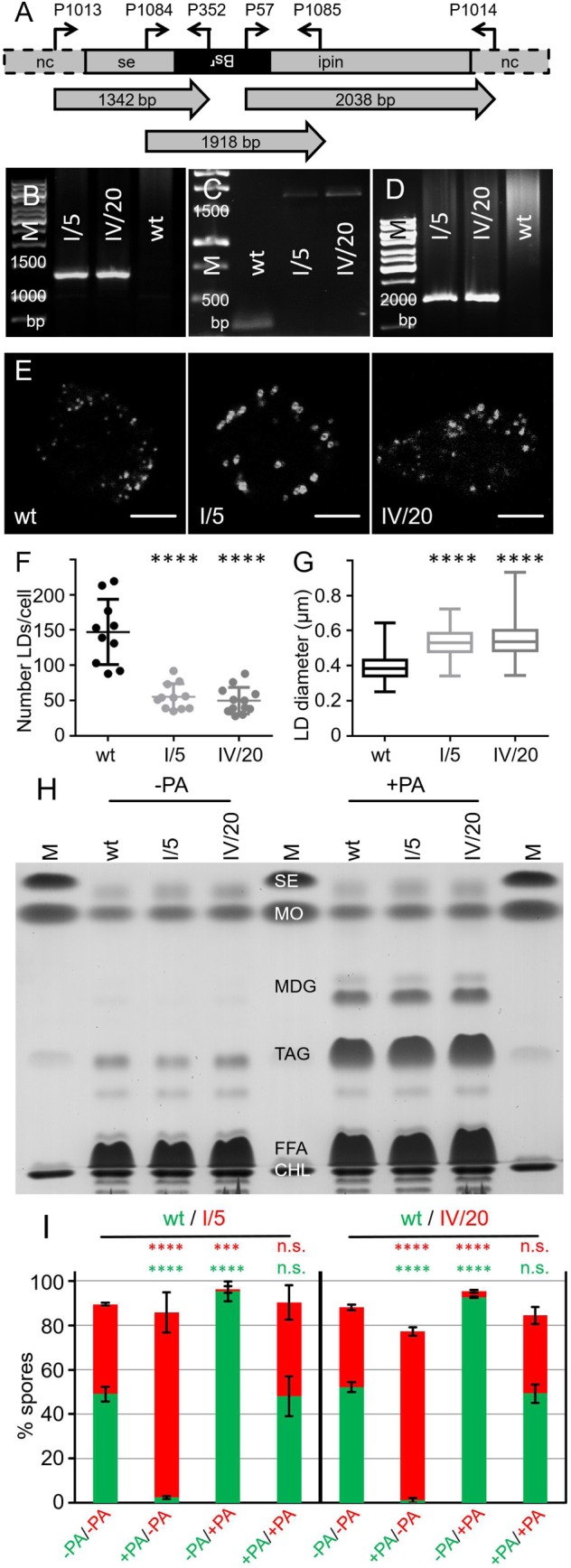
**Lack of seipin neither affects overall TAG production nor developmental fate.** (A) Diagram of the genomic *seiP* locus after insertion of a blasticidin S resistance cassette by homologous recombination in the coding region at the position corresponding to amino acid 244. Binding sites for diagnostic primers are indicated by arrows and reside in the coding region (seipin), the resistance gene (Bs^r^), or the noncoding regions (nc) upstream and downstream of the sequences used for targeting. The positions and sizes (bp) of diagnostic PCR products are indicated beneath. (B-D) PCR products from genomic DNA isolated from the wild-type strain (wt) and two independently derived *seiP^−^* mutants (I/5 and IV/20). (B) Combining one primer binding 5′ upstream of the *seiP* coding region (P1013) and one primer specific for the resistance cassette (P352), the disrupted copy of the *seiP* gene can only be amplified in the mutants but not in wild type. (C) Two primers situated in the seipin coding region (P1084 and P1085) amplify a 320 bp fragment from wild-type DNA, which increases to 1918 bp in the mutant strains, indicating the insertion of the Bsr-cassette concomitant with the absence of the original gene. (D) The primer pair P57 and P1014, placed in equivalent positions to panel B, but now with respect to the 3′end of the gene, also reveals the correctly sized diagnostic fragment of 2038 bp. The relevant sizes of the DNA marker (M) are given in base pairs. (E) Single optical sections through wild-type cells (wt) or *seiP^−^* mutants (I/5 and IV/20) supplemented for 3 h with palmitic acid and the fluorescent fatty acid tracer C_1_-BODIPY-C_12_ revealing reduced numbers of LDs in *seiP^−^* cells, as quantified in (F) concomitantly with increased LD diameter, as determined in (G). Scale bars: 5 µm. For (F) at least ten 3D stacks of each strain were analyzed for LD numbers, whereas 20 LDs each were used for the size measurement. ****P<0.0001 if compared to the wild type. Data are presented as mean±s.d. (H) Thin layer chromatography resolving SE, MDG, TAG, FFA, CHL, and MDG, from lipid extracts of wt and *seiP^−^* mutants incubated for 3 h with palmitic acid (+PA) or in normal medium (−PA). Abbreviations are as in Fig. 3E. Four biological replicates were made. (I) Bar diagram representing spore numbers after development of wild-type cells carrying an integrating plasmid for GFP expression (green bars) mixed with *seiP^−^* strains I/5 and IV/20 producing RFP from an extrachromosomal vector (red bars). These genetic properties explain the number of non-fluorescent cells (difference to 100%) that are slightly higher than in previous experiments. The cells that received palmitic acid for 3 h before the onset of development are marked by +PA. Otherwise, the 3 independent experiments were conducted and evaluated as described in Figs 2-4.

In order to address seipin's role in lipid droplet biogenesis, the corresponding mutant was constructed by homologous recombination (Fig. 6A). We inserted a resistance cassette into the strongly conserved loop (amino acid position 243) that is thought to point into the lumen of the endoplasmic reticulum (ER) and was suggested to be essential for seipin's function (Cartwright et al., 2015; Wang et al., 2016). Of the 48 clones tested, roughly one third carried the disrupted *seiP* gene. Two clones (I/5, IV/20), originating from independent transformation events, were preserved and their genetic makeup was verified by polymerase chain reaction (PCR) analysis (Fig. 6B,C,D). In *seiP^−^* mutants that were fed with a fluorescent fatty acid, it was immediately apparent that they contained fewer but larger lipid droplets as compared to wild-type cells (Fig. 6E). Supersized but fewer LDs are also a hallmark of seipin-deficient yeast cells (Fei et al., 2008; Szymanski et al., 2007; Wolinski et al., 2011), whereas smaller and more numerous LDs occur in fibroblasts from seipin patients (Szymanski et al., 2007), as well following siRNA knockdown of seipin, e.g. in HeLa cells (Fei et al., 2011).

A quantification of LD size and number in the *Dictyostelium* seipin mutants (Fig. 6F and G) enabled us to calculate the volume of the cell that is occupied by LDs and we arrived at a value suggesting that the *seiP^−^* mutants would store only 9% less fat than the wild type. Indeed, lipid extraction followed by thin layer chromatography confirmed roughly equal TAG amounts for wild-type cells and *seiP^−^* mutants (Fig. 6H). Finally, when *seiP^−^* mutants and the wild-type strain were labelled with cytoplasmic RFP and GFP, respectively, they developed into mixed fruiting bodies that contained equal amounts of spores (Fig. 6I). Each *seiP^−^* strain, when treated with palmitic acid, almost failed to contribute to the spore fraction (Fig. 6I), suggesting that the total volume (or mass) of fat which is accumulated by cells determines their success to proceed through development.

## DISCUSSION

### Do LDs affect cell differentiation?

Across various organisms, the literature provides interesting examples for LDs determining the cell fate. In mammals, more specifically in pigs, the earliest decision in development is when the blastomeres that contain more LDs form the embryo, while the ones with less LDs develop into extraembryonic tissue (Kim et al., 2012). More examples come from the *Drosophila* system, where maternal histones in the egg need to be sequestered on the LD surface to allow for unperturbed development of the early embryo (Li et al., 2014). Later in *Drosophila* development, wing imaginal discs accumulate surprisingly high levels of LDs that depend upon perilipin expression, which in turn is induced by a developmental transcription factor, vestigial (Fauny et al., 2005). If perilipin is knocked down, mitochondrial-derived reactive oxygen species accumulate in the wing disc, many cells undergo apoptosis, and the wing remains small and misshapen (Men et al., 2016). As a final example, *Drosophila* neuronal stem cells are maintained in a niche where the surrounding glia cells contain ample LDs to protect lipids from peroxidation reactions (Bailey et al., 2015). As the common motif in all these examples, LDs provide an advantage to the cells bearing them. In contrast, our results from the *Dictyostelium* model constitutes an example where LDs are disadvantageous, because fat cells do not contribute to the spore mass and are thus excluded from the next generation of amoebae.

One instance where this separation may occur is in the mound stage where we observed a concentric arrangement of lean cells in the middle and fat cells in the periphery (Fig. 2E). One possibility is that this sorting reflects some change in the efficiency of cell-cell adhesion, possibly involving molecules like csA, cadA and members of the tgr-family. Indeed, mutants affecting these proteins display reduced efficiency of spore formation (Ponte et al., 1998; Wang and Shaulsky, 2015; Wong et al., 2002). Because the cadA protein was also identified as a constituent of biochemically purified lipid droplets (Du et al., 2013), future investigations will address the possibility that sequestration of this protein leads to loss of adhesive function. Alternatively, the concentric arrangement may correspond to the stage when pre-stalk cells cluster together and move up in the periphery in a spiralling fashion to form the future tip of the slug (Clow et al., 2000), which later gives rise to the stalk and other cell types that fail to reproduce.

It has been known, for a long time, that the position a cell occupies in the slug is governed by its metabolic state. *Dictyostelium* cells grown in the absence of glucose sort to the tip of the multicellular migratory stage, and thus end up in the stalk; whereas cells that were provided with glucose predominate in the rear portion of the slug and subsequently become spores (Leach et al., 1973; Thompson and Kay, 2000). Interestingly, Leach et al. (1973) also noted that when cells grown on bacteria were mixed with cells grown in axenic medium, the latter became spores, whereas the former were suppressed in this developmental fate. Our present results can explain this early finding because bacteria as a food source promotes the accumulation of TAG and therefore LDs (Long and Coe, 1974; Matsuoka et al., 2003, and N. Pawolleck and M. M. unpublished observations), while axenic growth does not (Fig. 3) (Du et al., 2013). Furthermore, glucose is barely metabolized into TAG at normal medium concentrations (Fig. 3E), and added palmitic acid strongly dominates in the possible cell fate decision (Fig. 3F).

How does the metabolic state communicate with developmental fate? Clearly, reserves of energy and building blocks determine the speed of the cell cycle. As a consequence, fat cells could be enriched in a different phase than lean cells. Because cell cycle position was shown to be the most critical determinant for the decision of stalk versus spore formation in *Dictyostelium* (Chen and Kuspa, 2005; Maeda, 2011; Muramoto and Chubb, 2008; Wood et al., 1996), it is conceivable that a cell-cycle checkpoint could be the site where the metabolic situation is converted into a developmental decision, and we will test this possibility in further experiments.

### Do LDs shorten the cell's lifespan or even cause cell death?

Mammalian cells are especially sensitive to added palmitate, since it induces ER stress, elevates the levels of reactive oxygen species, and causes cell death (Brookheart et al., 2009), whereas oleate is tolerated well. The adverse effects of palmitic acid can even be relieved by adding oleate at the same time, possibly because it promotes palmitate esterification into TAG which is true for mesenchymal cells and their derived osteoblasts (Gillet et al., 2015), hepatic stellate cells and hepatic epithelial cells (Hetherington et al., 2016), as well as for insulin-producing pancreatic cells from rat (Plötz et al., 2016), supporting the common idea that LD-formation is solely a way of detoxifying free fatty acids. Even yeasts show a similar behaviour (Nguyen and Nosanchuk, 2011), whereas *Dictyostelium* cells respond in exactly the opposite fashion: added oleate immediately stops further cell division, acting over days, even if it was administered only once, whereas palmitate has no adverse effect on cell growth (Fig. 1I).

We must emphasize that it is specifically the presence of the lipid droplet that eliminates fat *Dictyostelium* cells from the next generation. In our model system, only the wild type or the *seiP^−^* mutant, that are both fully competent to synthesize TAG and to build LDs, fail to survive the competition experiment (Figs 2H,I, 4I and 6H,I) if challenged with palmitic acid in the growth medium. The *dgat1/2^−^* double mutant line that is still able to produce DAG, from which membrane lipids are derived that accumulate in the ER (Barisch and Soldati, 2017; Du et al., 2014), is not deficient in this respect (Fig. 4H,I). Also, the mutant lacking the FcsA protein, and therefore being largely incompetent to produce activated coenzymeA-linked fatty acids, can form a normal fraction of spores, even if cultivated in the presence of palmitic acid (Fig. 4H,I).

Neither in humans nor in experimental animals is the relation between fat storage and lifespan really clear. Low TAG levels originating from caloric restriction can shorten the lifespan of mice (Liao et al., 2011), while the same condition produced by genetic loss of the DGAT1 enzyme promotes longevity (Streeper et al., 2012). The same is true for *Caenorhabditis*, as it depends on the underlying cause of increased fat content, whether the animal lives for a long time (Steinbaugh et al., 2015) or dies early (Zarse and Ristow, 2008). Recent experiments in yeast have shown less ambiguously that an elevated TAG content, whether caused by inactivating the lipase or overexpressing DGAT, will result in an increased lifespan, whereas a DGAT knockout strain, where TAG is virtually absent, is rather short lived (Handee et al., 2016).

In the presence of palmitic acid, growth of *Dictyostelium* cells proceeds for several generations (Fig. 1I). Cells first build up lipid stores for about 3 h and then consume them over the next 24 h (Du et al., 2013). If the initial medium is exchanged for growth medium lacking palmitic acid, TAG consumption even speeds up (Du et al., 2013) and the cells remain healthy. In stark contrast, cells that were allowed to build up lipid droplets, which are then starved in non-nutrient medium, behave normally for roughly 8 h, but start to die when forming the aggregate, whereupon their corpses are phagocytosed (Fig. 2C). For one, it is interesting to observe that phagocytosis occurs so late in development, as it was previously thought to decline a few hours after the onset of development (Katoh et al., 2007). Secondly, phagocytosis of cell corpses from the same species is generally assumed to occur after these cells have undergone programmed cell death. In *Dictyostelium* one form of apoptosis occurs when development is blocked (Tatischeff et al., 2001), the other sort of cell death is of the autophagic type, but it has been only studied under conditions that are not compatible with multicellular development (Giusti et al., 2009). Thus it remains to be seen whether the cell death we observe, as being dependent on the existence of lipid droplets, bears characteristics of one or the other form.

The mammalian cell death-inducing protein CIDE-C, fails to induce apoptosis if bound to lipid droplets (Liu et al., 2009). Thus, we currently consider the possibility that an apoptosis inducing factor, AifC, which was co-purified with *Dictyostelium* lipid droplets isolated from vegetative cells (Du et al., 2013), could be liberated from LDs under a stimulus that is specific to the aggregation stage. This mechanism would explain that palmitic acid-induced cells die at individual time points during and after aggregation, and they are sorted to the periphery (Fig. 2E) and left behind when the slug or culminant is formed (Fig. 2F). Accordingly, we assume that the main reason for the dominance of lean spores developing from mixed populations is the death of fat cells and not only the change of their cell fate.

## MATERIALS AND METHODS

### Cell growth, mixing and development

Cells of the *Dictyostelium* AX2 strain (referred to as wild type) and mutants constructed in this genetic background were grown in HL5+ medium (Formedium, UK) in shaking suspension and lipid droplet induction was performed by adding fatty acid to a final concentration of 200 µM as described previously (Du et al., 2013). 6×10^6^ cells from each of two strains expressing two different fluorescent proteins (GFP and RFP) were mixed, and immediately harvested at 4°C for 5 min at 400 ***g***. To initiate development, cells were washed with Soerensen's phosphate buffer (2 mM Na_2_HPO_4_, 15 mM KH_2_PO_4_), resuspended and put on 5 ml phosphate agar plates, resulting in 6×10^5^ cells cm^−2^. After 24 h, fruiting bodies were collected and spores or germinated cells were analysed via fluorescent microscopy. For quantitative distribution, at least 100 spores or cells were counted. The statistical analyses were performed using Prism (GraphPad). Significance was determined via one-way ANOVA with Dunnet's test to compare the mean of each condition with the mean of the control experiment. The precision of mixing was estimated by calculating the mean value of green wild-type spores from all untreated samples in the experiments shown in Figs 2, 3, 4 and 6 yielding 51±3.3%.

### Molecular biology

DNA and protein sequences for *Dictyostelium seiP* (DDB_G0287697) encoding seipin were obtained from the fully sequenced genome (Eichinger et al., 2005) via http://dictybase.org (Kreppel et al., 2004), where they are also linked to studies of expressed sequence tags. Transmembrane regions were identified at http://ch.EMBnet.org. Because a PCR of full-length *seiP* failed, two fragments of the gene were ligated via an endogenous BglII site. The first fragment was amplified on cDNA created via reverse transcription (ThermoFisher Scientific) from total RNA (TRIzol, Invitrogen) with primers 996 (GCTTCTAGAATGGAAAATATAGTTAGTAAATC) and 995 (GCTAGATCTTTGGTGTTGACTTTCGAAATC) followed by ligation into pGEM-T Easy (Promega) creating plasmid 1224. For the second fragment, primers 992 (GCTAGATCTTTAACTTCTTCTCCTTTAATTAG) and 958 (GCTACTAGT TTTTCTCTTTCTTACTGAAGAA) were used to yield plasmid 1225. After digestion with BglII and SpeI the second fragment was ligated into vector 1224 opened with the same enzymes. The resulting XbaI and SpeI fragment was excised from plasmid 1226 inserted into pDM323 (Veltman et al., 2009) opened with SpeI, expressing seipin-GFP (1227). To link the N-terminal part of seipin comprising amino acids 1-411 to GFP, primers 961 (GCTACTAGTATGGAAAATATAGTTAGTAAATC) and 960 (GCTACTAGTAGATGATGATGATGATGATGTTG) were used on cDNA and the product was first inserted into pGEM-T Easy, generating plasmid 1131 from where it was transferred via SpeI into pDM323, yielding vector 1133. The same was done for seipin^412-753^ except that primers 962 (GCTACTAGTATGAATTTAAAAACCACATTTTTATCAAA) and 958 (GCTACTAGTATGAATTTAAAAACCACATTTTTATCAAA) were used, creating plasmid 1134 in pGEM-T Easy, and 1135 in pDM323. For disrupting *Dictyostelium seiP*, plasmid 1131 was cut with SwaI and via blunt end ligation the 1.6 kb blasticidin resistance (Bs^r^ ) cassette flanked by SmaI originating from pLpBlp (Faix et al., 2004) was inserted. After restriction with EcoRI the *seiP* fragment containing the Bs^r^ cassette was cut out and transfected into AX2 cells by electroporation. The resulting clones were tested via PCR for the correct homologues recombination of the construct with two primers binding in the Bs^r^ cassette (primer 57, CGCTACTTCTACTAATTCTAGA and primer 352, CGCTACTTCTACTAATTCTAGA), within the coding region (primer 1084, CAGCATCAATCCAAATTAATAATCCAGAG and primer 1085, GCCAACTAAAATATCATCATTTTCAGGTAC), as well as in the 5′ and 3′ UTR of *seiP* (primer 1013, CAATATTAACAATTCACCTAAAAATAG and primer 1014, CTTCCAAGAAAATCAAAAAAACTTG).

### Lipid analysis

The classical method of lipid preparation by Bligh and Dyer (1959) was adapted as described previously (Du et al., 2013). Subsequently, the lipids were separated by thin-layer chromatography (TLC) on silica plates using two solvents. When the first solvent front (hexane:diethylether:acetic acid 80:20:1) had reached two thirds of the separation distance, the plate was air dried and further developed in a second solvent system (hexane:diethylether 49:1) to completion. To visualize the lipids, the plates were stained for 3 s with copper sulfate (0.6 M in 8.5% phosphoric acid) and heated to 160°C for 15 min to conduct the charring reaction.

### Immunofluorescence experiments and GFP microscopy

Immunofluorescence experiments and GFP microscopy were performed as described before (Maniak et al., 1995). The distribution of the endoplasmic reticulum was shown by indirect immunofluorescence using undiluted mouse monoclonal antibody supernatant raised against the protein disulfide isomerase (PDI, MAb 221-64-1) (Monnat et al., 1997). Monoclonal antibodies were detected using CY3-coupled goat-anti-mouse polyclonal secondary antibodies (Dianova, Germany) or those labelled with Oregon Green 488 (ThermoFisher Scientific). The lipid droplet-specific dye LD540 (Spandl et al., 2009) was diluted from its stock (0.5 mg ml^−1^ in ethanol) to a final concentration of 0.3 µg ml^−1^ in phosphate-buffered saline (PBS) and used to stain fixed cells for 30 min instead of using an antibody. Alternatively, LDs were metabolically labelled with the fluorescent fatty acid analogue C_1_-BODIPY-C_12_ (500 nM final concentration, ThermoFisher Scientific) supplemented together with palmitic acid for 3 h in growth medium. Images were taken as single confocal planes or stacks using a Leica TCS-SP laser scanning microscope.
